# First report of *SYNE1* arthrogryposis multiplex congenita from Saudi Arabia with a novel mutation: a case report

**DOI:** 10.1186/s13052-022-01301-x

**Published:** 2022-06-23

**Authors:** Naglaa M. Kamal, AlaaEddin M. Alzeky, Maher R. Omair, Ruwayd A. Attar, Abdullah M. Alotaibi, Abdullah Safar, Nawal S. Alosaimi, Sara A. S. Abosabie

**Affiliations:** 1 Pediatrics and Pediatric Hepatology, Kasr Alainy Faculty of Medicine, Cairo University, Cairo, Egypt; 2 Alhada Armed Forces Hospital, Taif, Kingdom of Saudi Arabia; 3 Faculty of Medicine, Charité Universitätsmedizin Berlin, Berlin, Germany

**Keywords:** *SYNE1*, Arthrogryposis multiplex congenita type 3, Saudi Arabia, Novel

## Abstract

**Background:**

Myogenic Arthrogryposis Multiplex Congenita type 3 (AMC-3), is a rare congenital condition characterized by severe hypotonia, club feet, and multiple joint contractures often affecting both arms and legs which start prior to birth.

**Case presentation:**

We report a full-term neonate born to first-degree cousins from fourth-generation consanguineous families, who had with antenatal history of reduced fetal movements. At birth, he was noticed to have bilateral club feet, arthrogryposis, severe hypotonia, and absent deep tendon reflexes. The patient developed difficulty in breathing probably attributed to his generalized severe hypotonia, necessitating mechanical ventilation.

His creatinine-phospho-kinase, electromyogram, and brain magnetic resonance imaging were normal. Whole-exome sequencing (WES) was requested for the genetic diagnosis of the case.

WES identified a novel homozygous variant c.23415-3799C > G p. in the synaptic nuclear envelope protein1 [*SYNE1*] gene. Seven out of 20 bioinformatic in silico programs predicted a pathogenic effect for this variant. Segregation analysis of the variant in the parents and siblings revealed that both parents and one sibling were heterozygous for the same mutation which proved the variant significance and its autosomal recessive pattern of inheritance.

**Conclusions:**

AMC3 should be suspected in patients with decreased fetal movements, severe hypotonia, absent deep tendon reflexes, and arthrogryposis. *SYNE1* gene mutations can be the underlying genetic defect and molecular genetic testing can prove the diagnosis.

## Background

Arthrogryposis multiplex congenita (AMC), describes a variety of unprogressive skeletal defects with joint contractures presenting at birth and found throughout the body with arms and legs being more commonly affected [1].

Arthrogryposis in general is a rare condition with a prevalence of one in 3000 in the general population [2]. It is less common in the European population, occurring in 1 out of every 11,000 – 12,000 live births [3].

There is no single cause for AMC. Decreased fetal movement in utero is one known factor, which can occur due to a variety of reasons, including environmental factors (i.e. maternal illness, limited space), gene mutation (autosomal dominant, autosomal recessive, X-linked), chromosomal abnormalities, and various syndromes [4].

Myogenic-type arthrogryposis multiplex congenita-3 (AMC3) is a rather more rare type of AMC. It is an autosomal recessive disorder characterized by decreased fetal movements, hypotonia, variable skeletal defects, including clubfoot and scoliosis, and delayed motor milestones with difficulty walking [5].

SYNE1 gene is found in many tissues and encodes nesprin-1, a member of the spectrin family of structural proteins that link the nuclear plasma membrane to the actin cytoskeleton. Mutations in this gene have been associated with multiple congenital conditions, one of them is Myogenic-type AMC3 [5].

The present report describes a Saudi newborn baby boy with dysmorphic features, hypotonia, and multiple joint contractures, in whom Whole Exome Sequencing identified a novel homozygous variant c.23415-3799C G p.(?) in the SYNE1 gene (OMIM:608,441).

## Case presentation

SA is a 6-month-old Saudi male who was born to healthy first-degree cousins from fourth-generation consanguineous families. There was no history of maternal risk factors during pregnancy. His mother is a 30-year-old housewife with three apparently healthy offspring. Reduced fetal movements were reported by the mother during pregnancy and were detected by antenatal ultrasound scans. The patient was delivered at term by elective cesarean section due to breech presentation.

Apgar’s score was 5 and 7 at 1 and 5 min respectively. Birth weight was 2595 g (50th centile) and occipitofrontal circumference was 37 cm (50th centile). At birth, there was severe hypotonia and the baby was admitted to the neonatal intensive care unit with poor respiratory efforts which might be attributed to his generalized severe hypotonia. He required intubation with positive pressure ventilation. There was no family history suggestive of any neuromuscular diseases.

**Fig. 1:**
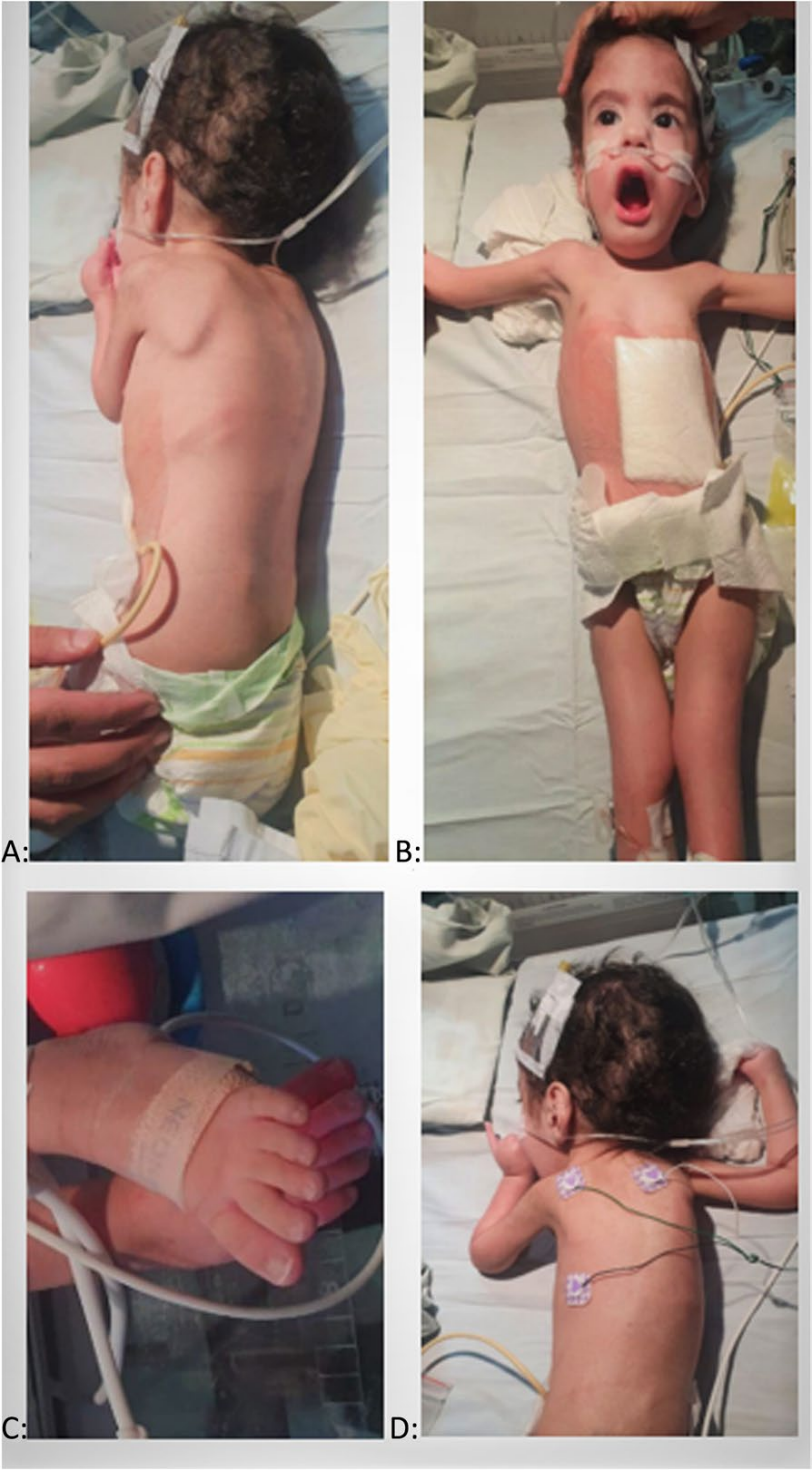
**a** Winging scapula. **b** Myopathic facies; elongated face, chest deformity, pectus excavatum, knee joint contracture. **c** Long slender toes. **d** Kyphoscoliosis

His examination revealed subtle dysmorphism in form of a long narrow face, long philtrum, depressed nasal bridge, flat occiput, and large ears. He had arthrogryposis multiplex in the upper & lower limbs, involving interphalangeal joints, metatarsophalangeal joints, thoracolumbar scoliosis, bilateral knee dislocation, bilateral club feet, clenched hands, and overlapping fingers (Fig. 1a-d).

Neurological examination showed generalized muscle wasting, and severe hypotonia. Mild facial weakness was observed without ophthalmoplegia. Deep tendon reflexes were absent. Neither pyramidal nor cerebellar involvement was noticed.

Abdominal examination showed no organomegaly and normal looking genitalia with bilateral undescended testis. His other body system review was unremarkable.

Laboratory investigations revealed normal complete blood count, renal and hepatic functions, normal electrolytes, and normal creatinine kinase as well as metabolic screening.

Echocardiogram showed a small mid-muscular ventricular septal defect and small patent ductus arteriosus with otherwise normal cardiac structure and function.

His electromyogram showed normal motor and sensory nerve conduction velocities with no other conclusive findings, and it was advised to be repeated after 6 months. Muscle biopsy was not done and Brain magnetic resonance imaging showed no abnormal findings.

Karyotyping was done and revealed a normal 46, XY male karyotype. Accordingly, whole-exome sequencing was requested.

### Molecular genetic analysis of whole-exome sequencing

**Fig. 2:**
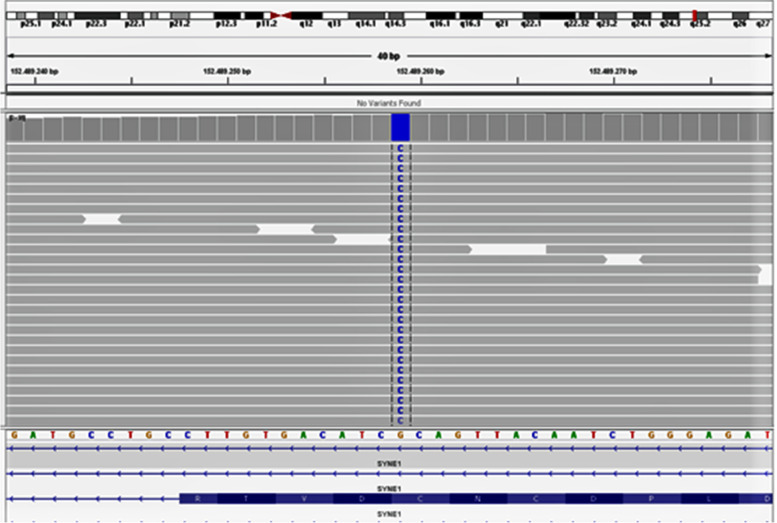
Whole-exome sequencing of the patient

Molecular genetic analysis of whole-exome sequencing was done (Fig. 2).

### Analysis

The coding exons of more than 20.000 genes of the patient DNA were enriched and sequenced. Filtering of the exome data targeted recessive, X-linked, and dominantly inherited diseases.

## Methods

Genomic DNA was fragmented, and the exons of the known genes in the human genome, as well as the corresponding exon–intron boundaries, were enriched using Roche KAPA capture technology (KAPA HyperExome Library), amplified, and sequenced simultaneously by Illumina technology (next-generation sequencing. NGS) using an Illumina system.

The target regions were sequenced with an average coverage of 154-fold. For about 100% of the regions of interest a 15-fold coverage, for about 90% a 20-fold coverage was obtained.

NGS data were aligned to the hg19 genome assembly. Variant calling and annotation were performed by the bioinformatics pipeline. Identified SNVs and indels were filtered against external and internal databases focusing on rare variants with a minor allele frequency (MAF) in gnomAD of 1% or less and removing known artifacts and variants in regions with highly homologous regions.

Classification of variants was conducted based on ACMG guidelines (Richards et al. 2015) considering database entries (incl. HGMD), bioinformatics prediction tools, and literature status. A change in pathogenicity classifications over time cannot be excluded. Variants annotated as common polymorphisms in databases or literature or that were classified as (likely) benign were neglected.

Putatively pathogenic differences between the wildtype sequence (human reference genome according to UCSC Genome Browser; hg19; GRCh37) and the patient's sequence were assessed using an established quality score. Variants not passing the quality threshold were verified using polymerase chain reaction (PCR) amplification followed by conventional Sanger sequencing.

The results were interpreted in the context of clinical findings, family history, suspected mode of inheritance, and other laboratory data.

## Results

A variant with a significant phenotypic overlap in the patient was identified (Table 1).

**Table 1:** Molecular genetic identification of the patient

Gene (Isoform)	Phenotype MIM number (Mode of inheritance)	Variant	Zygosity	MAF gnomAD [%]	Classification
*SYNE1* (NM_033071.3)	618,484 (AR) 612,998(AD) 610,743 (AR)	c.23415-3799C>G p.(?) chr6:152,489,259	Homozygous	0	Variant of Uncertain Significance

Keywords

Gene: Approved HGNC gene symbol

Isoform: RefSeq accession number of the reported isoform

Phenotype MIM Number: ID of the Online Mendelian Inheritance in Man® (OMIM®) disease entry

Mode of inheritance: Supposed mode of inheritance for the described condition

Variant; Nucleotide and amino add change end position as well as genomic position (hg19)

Zygosity: Variant zygosity

MAF gnomAD: Minor allele frequency in the gnomAD database in %

Classification: classification of the variant based on the ACMG recommendations

### Interpretation

WES identified the homozygous variant c.23415-3799C > G p.(?) in the SYNE1 gene on chromosome 6q25.2 (OMIM: 608,441). This is a deep intronic variant with an unclear consequence on the protein sequence. However, based on another isoform of the gene (NM_001347702.1) the variant nomenclature is c.81C > G p.(Cys27Trp) which leads to an amino acid exchange.

Seven out of 20 bioinformatic in silico programs predict a pathogenic effect for this variant. To the best of our knowledge the variant has not been described in the literature so far (HGMD 2020.2).

Allele frequency of this variant in the general population has not been documented and this is the first time to be reported. Considering the available information, the variant is classified as a variant of uncertain significance.

Pathogenic variants in the SYNE1 gene cause autosomal recessive myogenic arthrogryposis multiplex congenita type 3 (AMC3; OMIM: 618,484) among others. AMC3 is characterized by decreased fetal movements, hypotonia, variable skeletal defects, including clubfoot and scoliosis, contractures, and delayed motor milestones with difficulty walking.

Considering the homozygous variant in the SYNE1 gene and the matching clinical picture of the patient, the patient was diagnosed as myogenic arthrogryposis multiplex congenita type 3.

We requested a segregation analysis of the identified variant in the parents and other siblings to help in the further interpretation of the results and to improve variant classification and whether it was inherited in an autosomal recessive pattern or did it occur de novo? Analysis revealed that both parents and one apparently healthy sibling were heterozygous for the same mutation while the two other siblings were normal which proves the autosomal recessive inheritance of the disease.

Genetic counseling was offered to the parents and the possibility of having the same disease in future pregnancies. They were advised for close antenatal follow-up which allows detection of the condition early in the first three months of pregnancy. The option of in vitro fertilization with a selection of normal zygotes was also offered.

The patient was discharged home on gastrostomy tube feeding and non-invasive home oxygen support due to his feeding difficulties and intermittent need for oxygen support, respectively.

Clinic follow-up with multidisciplinary care teams involving pediatrician, pediatric genetics, pediatric neurologist, pediatric gastroenterologist, pediatric orthopedic, and physiotherapist were given.

## Discussion and conclusions

The term arthrogryposis, or AMC, refers to a group of nonprogressive disorders characterized by multiple joint contractures discovered at birth in the body [1].

Review of the Saudi literature, arthrogryposis was occasionally described but with different gene mutations and clinical syndromes. The mutation identified in this paper is a novel mutation that was not reported before from Saudi Arabia and has not been described in the literature so far. Reviewing English literature revealed only five reported cases with variable mutations in the *SYNE1* gene, with similar clinical manifestation [5–7].

Our patient is the sixth patient worldwide identified with an AMC-causing *SYNE1* mutation.

Other than AMC; all patients including our patient shared some clinical findings including reduced fetal movements, absent deep tendon reflexes, and severe generalized hypotonia. No pyramidal or cerebellar affection. There was neither polyhydramnios nor intrauterine growth retardation (Table 2). They had borderline to average intellectual growth and motor development is delayed without creatinine kinase elevation (Table 2).

**Table 2:** Summary of the clinical data of our patient and previously *SYNE1* AMC3 reported cases

**Clinical Data**	Patient 1 (our patient)	Patient 2 (1^st^ sibling in Attali et al. report) [7]	Patient 3 (2nd sibling in Attali et al. report) [7]	Patient 4 (Baumann et al. report)^5^
**Sex**	Male	Male	Female	Male
**Antenatal reduced fetal movements**	Yes	Yes	Yes	Yes
**Polyhydramnios**	No	No	No	No
I**nheritance**	AR	AR	AR	AR
**Dysmorphic feature**	Yes	No	No	No
**Skeletal**	Severe	Severe	Severe	NA
Spine	Scoliosis	Kyphoscoliosis	Scoliosis	NA
Clenched hands	Yes	NA	NA	No Only adducted thumb
Club feet	Yes	Yes	Yes	Yes
Joints dislocation	Yes	NA	NA	NA
Arthrogryposis	Yes	Yes	Yes	Yes
**Undescended testicles**	Yes	NA	NA	Yes
**Neurologic**				
Microcephaly	No	No	No	No
Severe generalized Hypotonia	Yes	Yes	Yes	Yes
Deep tendon Reflexes	Absent	Absent	Absent	Reduced
Epilepsy	No	No	No	NA
**Intelligence**	Normal	Normal	Normal	Low
**MRI brain**	Normal	NA	NA	NA
**EMG**	Normal	Normal	Normal	Normal
**CK level**	Normal	Normal	Normal	Normal
**Disease course**	Progressive	Progressive	Progressive	Progressive
**Outcomes**	Alive	Died at age of 22 years due to pneumonia and sepsis	Alive	Alive

*NA* data not available

There was very limited clinical data about the two siblings reported by Laquerriere et al.^6^ and they were not included in this table

The condition was progressive in one family with two siblings who were unable to walk at 12 years of age and developed severe scoliosis, leading to death from pneumonia in one of them at the age of 22 years [7]. There was no indication of cerebellar disease in our patient and previously reported patients. The life expectancy of the disease is not well known because five out of the six reported patients including our patient are younger than 20 years. Two reported sibs were reported in one paper with limited clinical details [6].

Molecular genetic testing proves the diagnosis and segregation analysis of the patients’ families helps for proper identification of genetic analysis results, zygosity and inheritance pattern. In our patient, his parents and one sibling were heterozygous for the same mutation.

Due to severe hypotonia, patients might need respiratory and feeding support. We inserted a gastrostomy tube to aid the feeding of our patient. He also needed home oxygen support.

Follow-up with multidisciplinary care teams is crucial to support the patient’s different medical needs. Genetic counseling of the parents for future pregnancies is a cornerstone to avoid having further affected children.

In conclusion; AMC3 is a rare disease. Physicians should suspect it in patients who have an antenatal history of decreased fetal movements, and who present at birth with arthrogryposis associated with severe hypotonia and absent deep tendon reflexes. Different laboratory and imaging studies are usually nonconclusive and molecular genetic analysis is needed to confirm the diagnosis. *SYNE1* gene mutation is a rare cause of this disease with only 6 reported cases including the current one.

## Data Availability

All data and materials related to the study are included in the current manuscript.

## Abbreviations

AMC: Arthrogryposis multiplex congenita
SYNE1: Synaptic nuclear envelope protein1
AMC3: Myogenic arthrogryposis multiplex congenital type 3
